# DAVE: A Comprehensive Software Suite for the Reduction, Visualization, and Analysis of Low Energy Neutron Spectroscopic Data

**DOI:** 10.6028/jres.114.025

**Published:** 2009-12-01

**Authors:** Richard Tumanjong Azuah, Larry R. Kneller, Yiming Qiu, Philip L. W. Tregenna-Piggott, Craig M. Brown, John R. D. Copley, Robert M. Dimeo

**Affiliations:** Center for Neutron Research, National Institute of Standards and Technology, Gaithersburg, MD 20899; Department of Materials Science and Engineering, University of Maryland, College Park, MD 20742; Laboratory for Neutron Scattering, ETHZ and Paul-Scherrer Institut, CH-5232 Villigen PSI, Switzerland; Center for Neutron Research, National Institute of Standards and Technology, Gaithersburg, MD 20899

**Keywords:** analysis, backscattering, inelastic, neutron, reduction, scattering, software, spectrometer, spin echo, time-of-flight, triple-axis, visualization

## Abstract

National user facilities such as the NIST Center for Neutron Research (NCNR) require a significant base of software to treat the data produced by their specialized measurement instruments. There is no universally accepted and used data treatment package for the reduction, visualization, and analysis of inelastic neutron scattering data. However, we believe that the software development approach adopted at the NCNR has some key characteristics that have resulted in a successful software package called DAVE (the Data Analysis and Visualization Environment). It is developed using a high level scientific programming language, and it has been widely adopted in the United States and abroad. In this paper we describe the development approach, elements of the DAVE software suite, its usage and impact, and future directions and opportunities for development.

## 1. Introduction

Low energy neutrons have been used to probe the atomic and molecular dynamics of condensed matter systems for more than 50 years [1]. Unfortunately, the community of researchers that uses neutron scattering has not settled on a single software suite for reduction, visualization, and analysis of neutron spectroscopic data. The lack of a single package can have negative consequences for user facility operations. For instance, when a facility commissions a new neutron spectrometer, new software will often need to be developed -sometimes reinventing an existing capability. This situation is due to a number of contributing factors including: (1) the lack of a coherent package that performs all of these essential tasks, (2) the lack of agreement on a software development platform (e.g., free vs commercial programming language) and, to a lesser extent, (3) the lack of a common data file format used by all of the facilities. Though the problem remains generally unresolved, we believe that the data reduction, visualization, and analysis product developed at the NIST Center for Neutron Research (NCNR) addresses many of these issues. In particular, we believe that the software development model adopted at the NCNR for the treatment of neutron spectroscopic data has been successful.

The NCNR is one of the top neutron research facilities in the country [2]. The Center supports important NIST measurement needs, but it is also operated as a major national user facility with merit-based access made available to the entire U.S. scientific and technological community. In fiscal year (FY) 2008, more than 2,200 researchers directly benefited from access to NCNR capabilities, accounting for over two-thirds of all neutron research performed in the United States. The NCNR is ranked as one of the highest impact neutron facilities in the world (with over 320 publications in FY 2008). NCNR users are a diverse group spanning undergraduate science and engineering students, graduate students, post-doctoral researchers. academic faculty, and industrial and government researchers. Users visit the facility in Gaithersburg, MD to carry out their neutron measurements which may take any length of time from a day to a few weeks. During this time the users need to be able to reduce and view their data in near real-time. Often it is necessary to go beyond data reduction and visualization to perform simple data analysis in order to better inform decisions for subsequent measurements. Users are diverse in terms of their experience with neutron scattering as a measurement technique. Though initially considered to be an *experts-only* technique, the last decade has seen a rapid increase in the number of non-expert users. Frequently the non-expert users require more attention from facility staff, especially in the area of treating the data and transforming it into a quantity that can be interpreted analytically. Our philosophy has been that a national user facility such as the NCNR has an obligation to provide easy-to-use tools to users to ensure a successful experiment, particularly with regard to data reduction.

In this paper we describe our effort to develop a comprehensive software package for the reduction, visualization, and analysis of neutron spectroscopic data as well as providing experiment planning tools. The result has been a widely used software package called DAVE (Data Analysis and Visualization Environment) based on the commercial software development language IDL^1^ [3]. DAVE is free for users to download from the NCNR website [4].

## 2. Neutron Spectroscopy

Neutron scattering is a powerful technique that is widely used to investigate the structure and dynamics of materials over wide ranges of length and time scales. In one type of experiment, commonly called neutron *diffraction*, a typically (though not always) monochromatic beam of neutrons strikes a sample, and the scattered intensity is measured over a range of scattering angles. To a good approximation this type of experiment yields the scattered intensity as a function of the momentum transfer $\vec{Q}={\vec{k}}_{i}-{\vec{k}}_{f}$ where ${\vec{k}}_{i}$ and ${\vec{k}}_{f}$ are the incident and scattered neutron wave vectors respectively. In a *spectroscopic* study the intensity is further determined as a function of the energy transfer, i.e., the change in energy of the neutrons on scattering, $\hbar \omega =E_{i}-E_{f}$, where *E*_i_ and *E*_f_ are the incident and scattered neutron energies respectively. Energy transfers in neutron spectroscopy experiments range from nanoelectronvolts (note that 1 eV ≈ 8066 cm^−^1) in studies of slow membrane shape fluctuations through micro-electronvolts in molecular reorientation and tunneling experiments to millielectronvolts in relatively fast diffusion and vibrational spectroscopy investigations.

In the present work we are principally concerned with the treatment of data obtained in neutron spectroscopy experiments. The interested reader is encouraged to consult any one of the standard references on neutron scattering for details of the technique and a discussion of its unique capabilities (see Refs. [5–7], for example).

The format and organization of the data obtained in a neutron spectroscopy experiment generally depend on the instrument, and the goal of data reduction is to produce a quantity (the double differential scattering cross section) which is (almost) independent of the instrument, dependent on the sample itself and on the strengths of the interactions between the neutrons and the sample’s constituent elements. When a single contribution dominates, this quantity is approximately, if not exactly, proportional to the corresponding scattering function (also known as the dynamic structure factor), $S(\vec{Q},\omega )$. An important exception to these remarks is that the quantity measured in neutron spin echo spectroscopy [8] is, with similar caveats, related to the Fourier transform of the scattering function.

In a typical spectroscopic measurement the data are broadened due to the effects of instrumental resolution [9]. In many cases the resolution function can be approximated as a shift-invariant lineshape (i.e., one whose shape does not change with energy). This then can be convolved with a theoretical lineshape for comparison with the measured data.

## 3. Evolution of the DAVE Package

### A. Motivation

In the 1980’s and 1990’s, when users came to the NCNR to perform an inelastic neutron scattering experiment, they found an ad-hoc collection of programs, most of which served a single purpose. There were many different command-line programs for performing simple data transformations and analyses. Few programs used a graphical user-interface (GUI) to facilitate simple operation. This state of affairs was due to a historically informal approach to software development—one that succeeded for many years due to the fact that neutron scattering was largely an *experts-only* technique with practitioners who were willing and able to devote the time necessary to develop their own programs. For years users developed their own reduction and analysis programs to suit their own needs. Typically, these programs were not particularly user-friendly, most were operated from a command line, and they were only used and supported by the developer for as long as he or she required.

By the year 2000, NIST was nearing completion of an expansion of its cold neutron inelastic measurement capabilities, and a number of new high-resolution inelastic neutron instruments were coming online. These included a direct-geometry disk chopper spectrometer (DCS), a high flux backscattering spectrometer (HFBS), and a neutron spin echo (NSE) spectrometer. One common theme among the instruments was that there was no agreed-upon software to be used for reduction, visualization, or analysis. Of these three software requirements, reduction is the most specific to a particular instrument whereas visualization and analysis are more general. What was missing at the time was a common manner by which all of the new inelastic spectrometers and the existing inelastic spectrometers could visualize and analyze data. Different methods for reducing the data were adopted on these instruments. On the DCS, a reduction application was developed using a combination of Tcl/Tk [10] and Octave [11] (an open source language that is mostly compatible with MATLAB [12]). On the HFBS, a small reduction program with a primitive GUI was constructed using IDL. Part of the commissioning of these instruments included the development of software to reduce and visualize the data. Since no single software package for reduction, visualization, and analysis existed for these instruments, a group of NCNR scientists decided to adopt a common programming language to develop such a package.

### B. The Development Approach

The approach adopted at the NCNR is a variant of the previously successful model in which neutron scattering experts on each instrument developed the code because they knew the most about the details of the measurement techniques, the spectrometers, and the user requirements. Each of these three factors is critical, but this approach loses its appeal as the number of nonexpert users increases. At the NCNR this approach was modified so that the instrument scientists, responsible for operating the instruments and assisting the users, were an essential part of the development process. As a result of this cooperation among the instrument scientists, a core group of software developers at the NCNR emerged to provide leadership, coordination, and support for the overall development of the package. This core group included two dedicated programmers with extensive neutron scattering experience. The DAVE development team that formed included this core team as well as numerous instrument scientists from the NCNR.

The decision of which programming tools to use was influenced by a number of factors. First, the resulting package had to be free to download for all NCNR users. This was and is important for users of a major user facility because many of them cannot afford to purchase a high-end software package. The development language also needed to have high-level programming constructs with a mature, sophisticated set of visualization and analysis routines that could be incorporated quickly into end-user applications. This point was important in order to keep the development barrier low to the instrument staff whose responsibilities also included operating the instruments and providing assistance to the users. We wanted to make it as easy as possible for instrument staff to develop software applications that would help them assist their users. Finally, the development language needed to have a mature toolkit of GUI elements in order to allow the construction of easy-to-use end-user applications. Provision of easy-to-use software is a key factor in enabling non-experts to carry out successful neutron scattering experiments. There were a number of candidate software development languages available. We decided to adopt a commercial platform because of the availability of mature development tools (primarily data manipulation and visualization) that were superior to freely available development languages at that time. However, we could not ask the users to purchase the commercial platform just to run the NCNR software. After investigating the modes of distribution available from a number of different commercial packages we found that the flexibility of IDL’s embedded license was best aligned at that time with the needs of the NCNR and its users. Using IDL’s distribution model, a binary executable file of the DAVE application is provided to users free-of-charge. This binary executable has an embedded IDL license offering the full capabilities of IDL necessary to run the DAVE software on the most popular operating systems: WINDOWS, MAC, and LINUX. A “beta” version of the software was released internally at the NCNR in order to identify and fix the most significant bugs. The first public “beta” release occurred in July 2002 via the DAVE website.

### C. Data File Format

A confounding factor for many neutron scattering software development efforts has been the inability to implement a data format standard. Though there has been a clear decision by the neutron scattering community to use the NeXus format [13], a successful global implementation of the format has been elusive. The first decision needed for DAVE was agreement on an internal data structure that would be common across the application suite. A data file format was specified in November 2001 that consisted of a hierarchical data structure and several functions that could be used to manipulate it.

### D. Education and Training

In order to disseminate common programming practices and to foster a coherent approach to software development for DAVE, several series of hands-on, practical IDL training courses were held at the NCNR. A set of training materials [14] that provides a basic understanding of IDL data manipulation functions, programming constructs, visualization capabilities, and analysis functions was developed. In addition, information specific to the DAVE software package was included in the course. In particular, the internal data format for DAVE was documented as well as the manner by which developers could include their own stand-alone IDL applications in DAVE. For the first few years, the IDL and DAVE development courses were taught multiple times per year at the NCNR and enrollment was open to all NCNR staff that were interested. A typical class had 12 attendees and demand has remained high. Students and faculty from academic institutions and staff from other neutron facilities (foreign and domestic) also attended these courses. This effort to train instrument staff paid off as evidenced by the rapid growth of the DAVE package. In some cases an instrument scientist who had taken one of the courses was immediately able to begin working on an end-user application.

## 4. Capabilities of the DAVE Package

The capabilities of the DAVE software package can be divided into four parts, and these parts align roughly with the sequence of events that take place in a neutron scattering experiment. These include a set of experiment planning tools, reduction programs for the different inelastic spectrometer instrument classes, visualization tools for all inelastic data types, and a set of basic and advanced data analysis tools.

### A. Experiment Planning Tools

There are numerous program modules in DAVE that are dedicated to helping users plan their experiments. Due to the limited time available for each measurement and because each spectrometer is unique, these tools can be very valuable in the decision-making process for the user when deciding on optimal sample dimensions and acceptable instrument parameters. Examples of these experiment planning tools are described next with a more complete listing in Table 1.

#### 1. TAS Scan Mapper

TAS Scan Mapper is a tool that assists triple axis spectrometer (TAS) users who wish to identify regions of reciprocal space that are inadvertently accessed during a scan. A scan is a set of instrument settings (angular positions of the monochromator, the analyzer, and the sample) that define points in (*Q*, *ω*) space to be accessed sequentially. At each point of a scan, counts are accumulated for a fixed number of counts measured by the incident beam monitor.

**Fig. 1:**
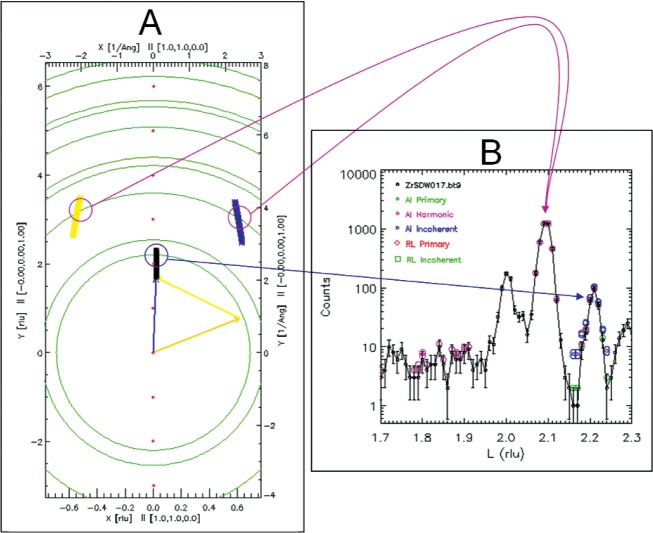
An example of TAS Scan Mapper output. Panel A depicts the scattering plane defined by orientation vectors [1,1,0] and [0,0,1]. Black symbols represent a longitudinal elastic scan from (0.02,0.02,1.7) to (0.02,0.02,2.3); the orange, yellow. and blue vectors show the incident and scattered wave vectors and the wave vector transfer, (i.e., ${\vec{k}}_{i}$, ${\vec{k}}_{f}$, and $\vec{Q}$), respectively, for the first point of the scan. Unwanted additional scans of wave vector transfers associated with second order scattering off the monochromator and off the analyzer, $2{\vec{k}}_{i}-{\vec{k}}_{f}$ and ${\vec{k}}_{i}-2{\vec{k}}_{f}$ respectively, are shown as sets of yellow and blue symbols, respectively. For clarity, all other wave vector transfers associated with higher order scattering have been omitted. Reciprocal lattice points for the sample and Debye-Scherrer rings associated with powder Bragg reflections from aluminum are shown. Panel B shows a plot of measured intensity versus the *z* component of $\vec{Q}$, labeled L and plotted in reciprocal lattice units. The experimental data are shown as black circles with error bars, and potentially spurious features are highlighted in colors based on their sources. The blue and purple curved connecting arrows relate points in the unwanted scans (as they cross aluminum powder rings) to corresponding features in the data. The feature at L = 2 is a Bragg peak from the sample.

It is unfortunate, but inevitable, that additional points in (*Q*,*ω*) space are visited unintentionally during a triple axis scan, for several reasons. These reasons include higher order reflections from the monochromator and/or analyzer, incoherent scattering from the monochromator or analyzer, and scattering from materials in the beam in addition to the sample. TAS Scan Mapper identifies these additional points and displays them as corresponding additional scans in the scattering plane. These unwanted scans occasionally generate so-called spurious peaks [15]. TAS Scan Mapper also displays a separate plot of the scans as a function of energy transfer. This is important because the energy transfers associated with the additional scans may cross the elastic condition, *ω* = 0, resulting in intensities comparable with intensities in the user’s intended inelastic measurement. Finally, TAS Scan Mapper displays experimental data as a function of a user-selected variable, highlighting points where spurious features are likely to occur within user-specified *Q* and *ω* tolerances. Fig. 1 shows a simple example.

#### 2. Neutron Cross Section Calculator

**Fig. 2:**
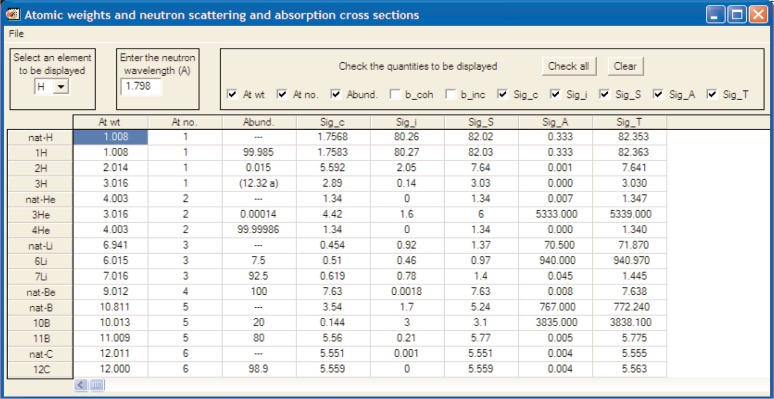
Neutron Cross Section table of the elements [16].

The cross section calculator is another useful planning application. Given a material’s chemical formula, its bulk density (or crystallographic density and packing fraction), and an incident wavelength, the number density (in formula units per unit volume), and the microscopic and macroscopic scattering and absorption cross sections per formula unit are calculated using published cross section information [16] that can also be viewed in DAVE as shown in Fig. 2. Relatively complicated formulae, such as that of the molecular magnet (*C*_2_*D*_5_)_2_*NH*_2_*Cr*_7_*NiF*_8_(*O*_2_*CC*(*CD*_3_)_3_)_16_, are readily handled. There is also a provision to include isotopes and isotopic mixtures. For example 85 % enriched uranium hexafluoride (^235^*U*_0.85_^238^*U*_0.15_*F*_6_) is represented as “\235U0.85\238U0.15F6”. The calculated cross sections are subject to important caveats, with particular regard to hydrogenous materials [17], displayed as a disclaimer.

#### 3. DCS Experiment Planner

**Fig. 3:**
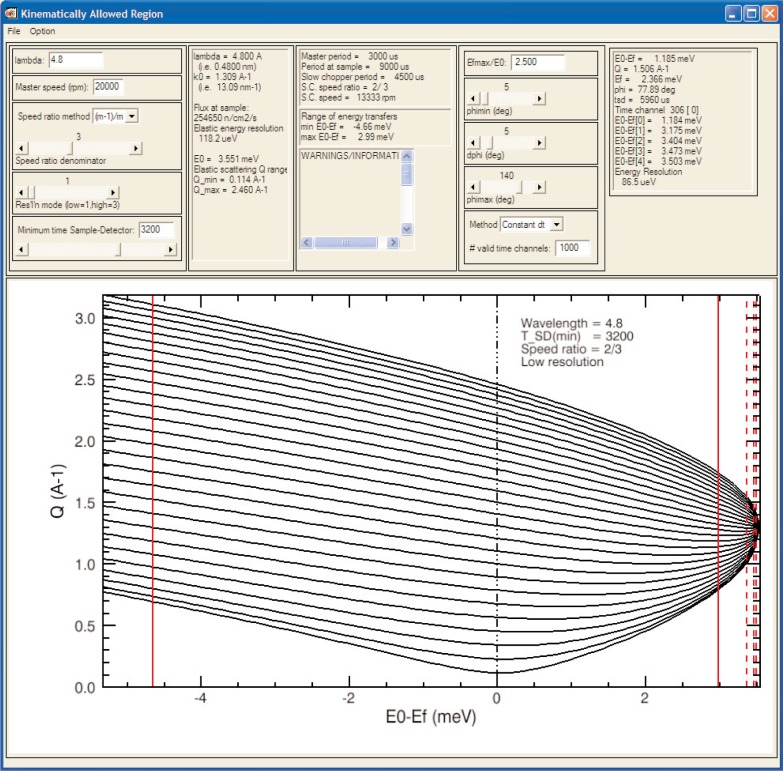
A plot, obtained using the DCS Experiment Planner, showing the kinematically allowed region bounded by heavy lines representing detectors at 5° and 140° and by solid red vertical lines representing the proposed range of times of flight from the sample to the detectors.

A planning tool specific to the DCS [18, 19] helps users decide the incident wavelength, the speed of the pulsing and monochromating choppers, and three additional quantities that determine the spectrometer’s coverage and performance in (*Q*, *ω*) space. Estimates of intensity at the sample and the (*ω*-dependent) energy resolution are displayed together with timing information inside and outside the covered region. Figure 3 shows a screenshot of the tool.

DAVE also includes tools to calculate self-shielding factors for slab-shaped and cylindrical/annular samples when there is no container and analogous quantities when there is a container. A good example is the factor that accounts for the reduction in the intensity of scattering by a container, due to the container itself and to the sample, for a typical measurement. The annular geometry tool can be used for multiple annular geometry, and it also calculates, as a function of scattering angle, the ratio of double to single scattering intensity using the isotropic elastic scattering approximation.

### B. Data Reduction

Neutron scattering instruments can be classified into categories in terms of their capability and method of operation. However, even within these categories, each instrument is unique since it is optimized to perform a specific task well or to cover a limited dynamic range. The output generated by an instrument (neutron counts and associated metadata) is referred to as the raw data and is unique to that instrument. Usually, it is necessary to convert these raw, instrument-specific, data into a quantity; the double differential scattering cross section, that largely depends on the physics of the sample under investigation, as described in Sec. 2. Since the instruments are unique, the corrections that must be applied to the raw data are instrument-specific. Hence, each instrument requires its own data reduction program module. Currently, DAVE data reduction covers the following five instrument classes: triple-axis, time-of-flight [17], filter analyzer [20], back-scattering [21], and spin echo spectroscopy [8, 22]. Specifically, DAVE supports eight instruments at the NCNR, two instruments at the Swiss Spallation Neutron Source (SINQ) at the Paul Scherrer Institut (PSI) in Switzerland, one instrument at the ISIS Pulsed Neutron Source at the Rutherford-Appleton Laboratory in the U.K., and one instrument at the Institut Laue-Langevin (ILL) in France. See Table 2 for a complete listing of currently supported instruments.

### C. Data Visualization

Data visualization tools enable the user to generate custom views of experimental data and make comparisons among different data sets. A good visual representation of, sometimes complicated, data enables one to immediately take in complex information, do basic qualitative analysis, or even arrive at a preliminary result. Visualization tools are particularly useful (during an experiment to help optimize the use of the beam time. The importance of visualization underscores the reason that a high-level, scientific programming language was selected as the development platform. Almost every program module provides some form of visualization, even if it is very basic. Several DAVE modules have a substantial visualization component, and these are highlighted next.

#### 1. Data Browser

**Fig. 4:**
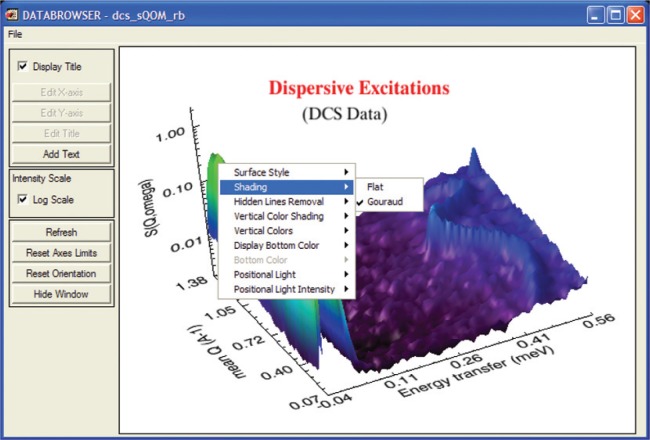
Surface view of a 2D data set with the Data Browser. Almost every attribute of the visualization can be customized. The excitation parameters can be determined by fitting the data, using the PAN analysis module.

The Data Browser is the one application module in DAVE that is mostly dedicated to visualization. While it also includes many data manipulation functions, these are included to support or enhance the data visualization capability. The Data Browser is designed to support DAVE formatted data, and multiple data sets can be loaded and managed simultaneously. Any number of views of one or more data sets can be generated from a single mouse click. The user can create multi-line plots from 1D (one independent variable) data sets and area (contour, image or surface) plots from 2D (two independent variable) data sets. The plots are fully customizable, and attributes such as symbols, colors, fonts, labels, legends, color tables, etc., can be altered. Basic data operations include: data rebin and rescale, generation of 1D slices from 2D data, and combination of multiple data sets into a single 2D data set. For improved use- ability, context-sensitive menus are used whenever possible as well as other mouse interactions with graphics such as rubber-band zooming and rotation. Also, a document-view architecture means that all views are automatically updated whenever the data they depend on changes. A typical surface plot produced using the Data Browser is shown in Fig. 4.

#### 2. Mslice

**Fig. 5:**
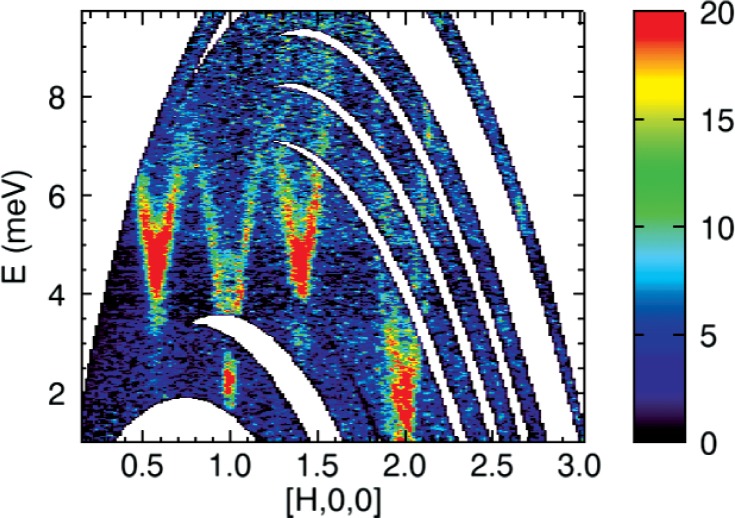
Excitation spectrum of a *URu*_2_*Si*_2_ single crystal in the H0L plane at *T* = 1.5 K, where L is integrated from −0.12 to 0.12. Note the minima at the antiferromagnetic zone center (100) and incommensurate positions (1 ± 0.4, 0,0). The feature at (200) is due to phonons. The color bar shows the intensity range [28]. This plot was generated using Mslice from multiple data sets taken at nine different orientations of the sample.

The program to visualize and analyze time-of-flight multi-detector instrument data is Mslice. Inspired by a program written in Matlab [27], Mslice in DAVE has several new features that allow data from new types of measurements conducted on time-of-flight instruments such as DCS to be directly visualized. In Mslice, data reduction is seamlessly performed for DCS raw data files. The scattered intensity as a function of scattering angle and time-of-flight, *I* (2*θ*, *t*), is converted to a weighted scattering function, $S(\vec{Q},\omega )$ or to a related quantity such as the generalized density of states. Corrections for detector efficiencies, sample self-shielding, and background subtraction can also be applied. Data sets from instruments other than the DCS are also supported, including data sets in the partially reduced SPE (intensity as a function of scattering angle and energy transfer) file format which includes the detector information file. Whereas for powder samples only the magnitude $\left|\vec{Q}\right|$ is necessary, for a single crystal sample, a projection into a 2-dimensional reciprocal space corresponding to the instrument scattering plane is carried out. For experiments that require measurements with the sample in multiple orientations, Mslice generates a composite visualization of the data files. In Fig. 5, a single crystal sample was measured at nine different sample rotation angles and the data files were loaded and visualized simultaneously. Each curved stripe represents one sample orientation angle in the measurement. The ability to aggregate multiple files taken at different sample orientation angles has significantly expanded the types of experiments that can be routinely performed on DCS. This is especially true in the field of magnetism. The program also provides useful tools for experiment planning and for analyzing instrument calibration files. One such tool is used to calculate and display the proposed detector trajectories in reciprocal space and the energy transfer before the experiment is performed.

#### 3. DenPro

DenPro is a 3D visualization tool for examining the results of crystallographic measurements. Given atomic coordinate positions and unit cell information in a crystallographic information file (CIF), DenPro displays spacefill and ball-and-stick models of the specified atoms. Currently DenPro supports the CIF format, and other formats may be added in the future. DenPro also displays the electron density or neutron scattering length density obtained in a crystallography experiment. The density data are specified as a 3D volume array within the unit cell, and DenPro displays the information as a 3D isosurface at a threshold value specified by the user. Currently DenPro supports the GRD file format [29] for reading in electron density or neutron scattering length density information.

**Fig. 6:**
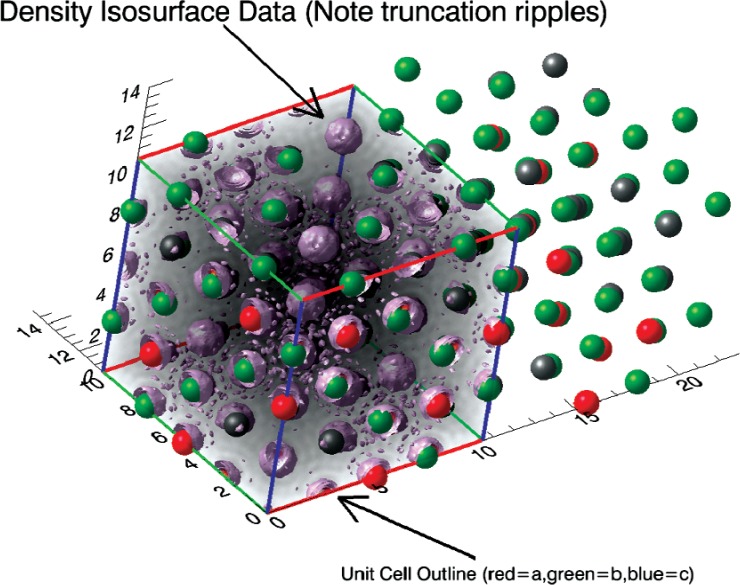
Visualization of the Fourier density map (*F_obs_*) and corresponding atomic positions for a simple spinel structure measured by single crystal x-ray diffraction.

DenPro takes advantage of the iTools framework of IDL. This framework provides functionality for annotation, full control of all 3D graphical elements, and positional and rotational control of the view. Such control of graphical elements allows features such as positioning multiple unit cells to better examine and compare crystallography results, as seen in the example in Fig. 6.

#### 4. Gaussian Viewer (G3dview)

After measuring a spectrum using the FANS instrument [20], it is typically desirable to compare the results with optical spectra and with the results of a molecular calculation to be assured of the correct assignments and symmetries for the modes. Optimization and normal-mode calculations of molecular fragments within semi-empirical, density-functional theory (DFT), Hartree-Fock, and higher-order levels of theory are routine, and DAVE provides a turn-key solution to compare measurements with calculations obtained from the Gaussian [30] software package.

The current version of DAVE supports the versatile and widely used Gaussian 03 (and Gaussian 98) packages [30] from which a geometry optimization and normal mode analysis is determined. Upon loading the Gaussian output file within the G3dview module of DAVE, the molecular structure is reconstructed, and the normal-mode eigenvalues and corresponding eigenvectors are read. A mass *m*_i_ and incoherent cross section *σ*_i_ are assigned to each atom i, with options to override the default values to allow for variations in the isotope concentration. The intensity of the j’th vibrational mode is expressed as follows (cf. Ref. [5], p. 56):

$$I_{j}=\sum_{i=1}^{N}(\sigma_{i}/m_{i})\exp(-2W_{i})e_{\text{ij}}^{2}$$ (1)

where *N* is the number of atoms in the molecule, *W_i_* is the Debye-Waller coefficient, and *e_ij_* is the associated eigenvector.

**Fig. 7:**
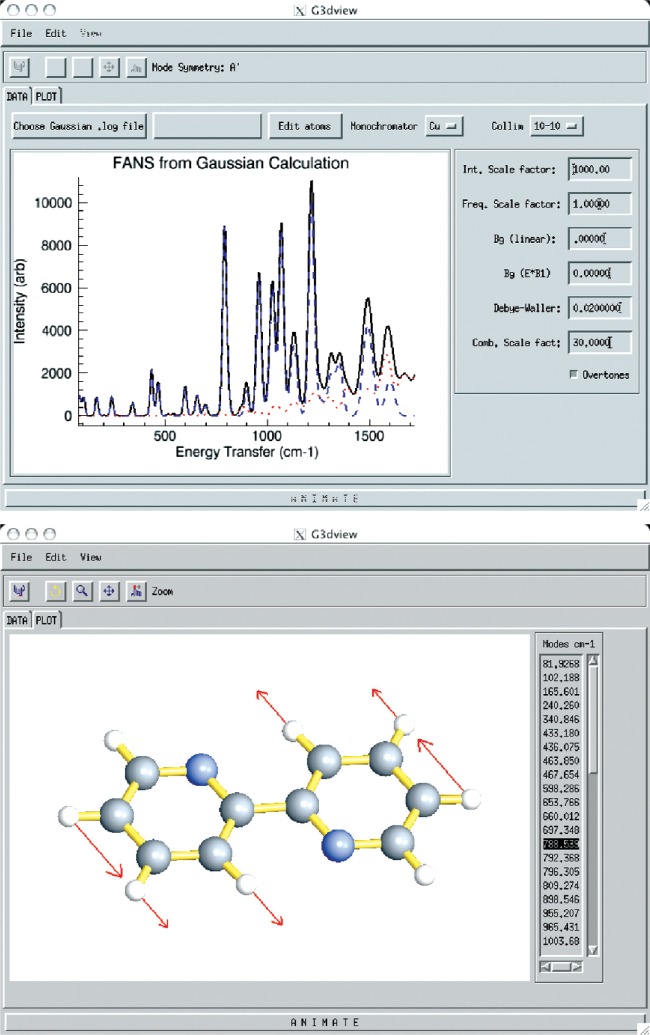
(Upper) Output from a Gaussian frequency calculation is used to model the predicted vibrational densities of states using the FANS spectrometer with instrumental resolution defined by the monochrornator and collimation conditions. The calculated curve (solid black line) can be compared to real data and empirically adjusted for factors such as background and vibrational overtones. (Lower) Animation and output of the vibrational modes are readily available with the click of a tab button. Relative atomic displacements for the selected eigenvalue are indicated by arrows in static figures.

The default spectral plot is the calculated fundamental frequencies convolved with the instrument resolution. The calculated spectrum can be adjusted on-the-fly for instrument resolution via the choice of monochromator and collimator reflecting the experimental conditions available on the FANS spectrometer. To account for differences in scale-factors and background contribution between calculations and data, user-defined input values are available to optimize the comparison. Another option allows the phenomenological addition of overtone bands to the spectrum while the use of object graphics allows for substantial customization of the resulting graph as shown in Fig. 7.

To further aid in visualization of the vibrational modes, a notebook tab displays a 3-dimensional view of the equilibrium molecular structure with customizable features for the atom and bond sizes, colors, transparency, and molecular position and orientation within the window. The modes can be animated and a series of images saved for importing into a presentation or combining into a movie for illustration purposes.

### D. Data Analysis

#### 1. Curve Fitting

DAVE includes a general-purpose curve-fitting application called PAN (for Peak ANalysis). Typically this application allows the user to fit a model function, *I*_fit_(*ω*), made up of one or more pre-defined lineshapes, to a measured data set. PAN includes a library of pre-defined functions such as the Gaussian, the Lorentzian, the Kohlrausch-Williams-Watts function [31], and several other functions, including one that can be used to model the background component of the data. If users wish to add their own function, there is an option to define a function and save it for later use. The model function is fitted to the data using an IDL adaptation of MINPACK, a robust set of non-linear least-squares fitting routines [32, 33].

**Fig. 8:**
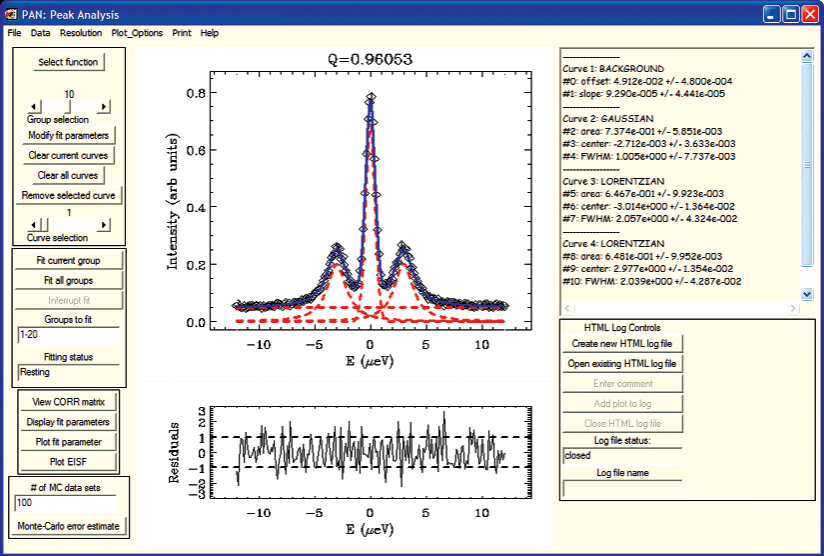
Using PAN to fit tunneling data taken on HFBS. The model function is constructed from a single Gaussian representing the central peak and two Lorentzians representing the inelastic peaks.

In order to make this application suitable for a broad audience and make it as simple to use as possible, extensive use was made of IDL’s widget toolkit. For example, when a function is added to the model the user can provide initial guesses for its fitting para meters (e.g., peak location, width, and intensity) via mouse interactions that provide immediate visual feedback. Often multiple data groups are read in simultaneously, and frequently variation between adjacent groups is relatively small. PAN allows the user to provide an initial guess for a single group of data and then it will automatically fit all of the data groups in succession using the results of the previous group as initial guesses for the current group. For simple cases it is not uncommon for a complete data set to be fitted in a matter of seconds. As discussed in section II, the instrumental resolution function should be factored into the data analysis. The instrumental resolution function can be obtained from another measurement or from an analytical model and then included in the analysis. The fit function is then written as:

$$I_{fit}(\omega )=\int d\omega^{\prime }I_{model}(\omega^{\prime })R(\omega -\omega^{\prime }),$$ (2)

where *I_model_* (*ω*) is the user specified function, and *R*(*ω*) is the shift-invariant resolution function. A typical session in PAN is displayed in Fig. 8.

Another analysis tool called RAINS (for Refinement Application for Inelastic Neutron Scattering) is more advanced. Whereas PAN fits two-dimensional data sequentially (e.g., different *Q*’s), RAINS performs a two-dimensional surface fit of *S* (*Q*, *ω*) to a data set.

The user selects a theoretical model with adjustable parameters to model the dynamic structure factor. Many of the models found in the classic neutron scattering texts by Bée [34], Press [35], and Hempelmann [36] are incorporated into RAINS. The instrumental resolution function is incorporated into the program in the same manner as in PAN.

#### 2. MagProp

**Fig. 9:**
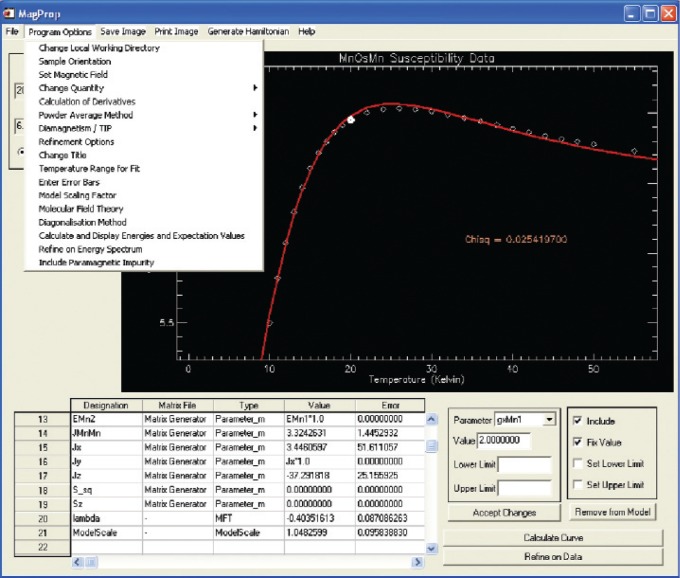
The main interface of MagProp. The program allows for the visualization and analysis of magnetic data. Experimental (symbols) and fitted (line) magnetization data for a molecular magnet are displayed.

The renaissance in magnetochemistry over the past decade may be broadly attributed to two factors. The first is the discovery of compounds exhibiting new physics such as slow relaxation of the magnetization on a molecular level [37]. The second is the emergence of inelastic neutron scattering and high-field Electron Paramagnetic Resonance (EPR) as complementary techniques, which can define the low energy magnetic excitations [38]. Thus, magnetochemists who perform inelastic neutron scattering experiments at neutron scattering facilities often wish to analyze their data in conjunction with magnetic data collected at their own laboratory. MagProp was written for this purpose. Magnetic data collected on home-built or commercial magnetometers may be readily formatted and displayed. The program reads in an analytical expression comprising the Hamiltonian and then generates the matrices from which the magnetic properties are calculated. Magnetic data may be refined to the model in conjunction with the excitation energies determined from spectroscopic techniques as shown in Fig. 9. The capabilities of the program are currently being extended to calculate INS and EPR spectra.

## 5. Usage and Impact

**Fig. 10:**
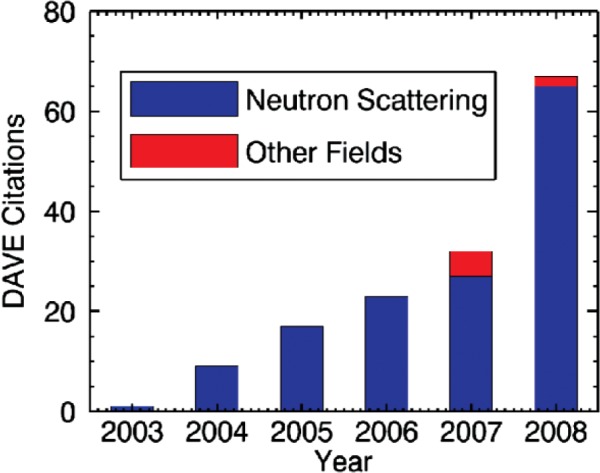
The number of citations of the DAVE software since its initial release. The data also show that DAVE has begun to be used in disciplines other than neutron scattering.

DAVE has become very beneficial to the users of the NCNR and the neutron scattering community. Many users, while operating the instruments at the NCNR and other facilities, use DAVE to quickly view their reduced data and perform simple analyses that inform decisions about their experiments. Formal acknowledgment within publications that have used the software is shown in Fig. 10. Since its initial release in 2002, DAVE has been adopted nearly exclusively by the users of the inelastic neutron scattering instruments at the NCNR. The first publication appeared in 2003, and publications have since grown significantly every year.

DAVE has also enhanced education and outreach activities at the NCNR. Assembling a large suite of related software has laid the foundation for introducing young scientists and new facility users to the variety of techniques available at the NCNR. The common look and feel among DAVE modules reduces the overhead necessary for both mature and novice neutron scientists to become comfortable with its various capabilities. The impact of DAVE is especially apparent at the Methods and Applications of Neutron Spectroscopy Summer School held every other year at the NCNR [39]. DAVE helps reduce the barrier to data treatment so that students from diverse educational backgrounds and levels can focus on the theoretical and experimental aspects of neutron scattering. For instance, several experiments in the Summer School have required interpretation of scattering data based upon quantum mechanical dynamics of molecules. As one example, the tools available in DAVE help the students easily calculate the rotational energy levels for quantum diatomic rotors, methyl rotors, and coupled methyl rotors. As useful as this simple number crunching is, with the graphical power of DAVE students can move to actually visualizing [40] what is happening to the wave packets through scattering events. This greatly aides the education process and fosters a deeper understanding of the physics behind the measurements.

With a relatively limited number of neutron scattering facilities in the world, scientists in the community tend to perform experiments at many of them. In addition to being widely used at the NCNR, DAVE has been adopted by other neutron scattering facilities. Despite the differences in neutron scattering instruments and the differing data file formats, other facilities use DAVE because they have similar data visualization and analysis needs. The well documented internal data format facilitates the adoption of the DAVE suite by instrument scientists. To date the following five neutron scattering facilities have partnered with the NCNR in developing DAVE software: the High Flux Isotope Reactor and the Spallation Neutron Source in Oak Ridge, TN; the OPAL reactor in Lucas Heights, Australia; the ISIS Spallation Neutron Source at the Rutherford Appleton Laboratory in the UK; and the SINQ neutron source at the Paul Scherrer Institut in Switzerland.

Looking at Fig. 10, it is interesting to note that the use of DAVE extends beyond scientists performing neutron scattering experiments. In the past few years, a number of astrophysics publications have cited use of the general-purpose analysis module, PAN. A detailed description of how PAN was modified to permit processing of emission line data from galactic outflowing gases can be found in a recent astrophysics Ph.D. thesis [41].

## 6. Future Development

DAVE evolved at a fast pace in its first three years, experiencing tremendous growth in the number and quality of modules available. Instrument scientists and other developers added programs and tools that they imagined would be useful to users. End users were also active participants in this growth by providing feedback and feature requests. The program modules were typically independent in most aspects especially with regard to design. implementation, and even the user interface. While aggregating modules into a single application suite was a significant step forward, there was little integration. In addition, the large size of the code base and the inevitable duplication of code meant it was becoming increasingly resource intensive to extend and maintain. We therefore decided to undertake a major overhaul of the application.

The main objective of the redesign was to create a more integrated user experience with a data-centric interface. better introspection capability, and simultaneous manipulation of multiple data sets. The new code base, DAVE 2, is largely based on IDL’s iTools Framework, which is a set of classes (abstract data types) with a well-defined application programming interface (API) that can be used to write applications with extensive built-in functionality. iTools is essentially a component framework that makes extensive use of object oriented programming. DAVE 2 consists of the following key elements: (1) A user interface consisting of a data manager, a visualization manager. and a visualization editor which together provide an environment in which the user can load multiple data sets simultaneously and can easily explore the contents (data plus metadata). (2) Sessions that can be saved into a project file and resumed subsequently; the save action preserves all data and application state and provides the possibility of working on multiple experiments. (3) An improved document-view architecture with superior standard visualization types and ubiquitous messaging (communication between components). (4) Data manipulation features that are encapsulated into components known as operations. Operations are well defined components that can be used to modify data, visualizations, or the application state. Usability has been significantly enhanced with multiple levels of undo/redo functionality for all core operations.

**Fig. 11:**
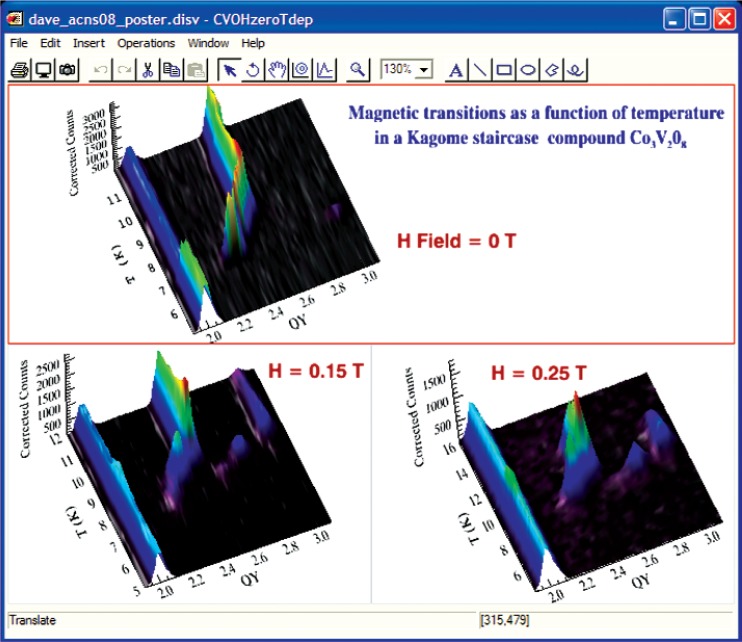
The visualization interface in DAVE 2 showing a customized window layout and text annotations. The data were taken on BT7 and were reduced using the TAS Data Reduction.

Users should benefit from the new functionality in a much better integrated application. For developers, the underlying component framework makes it easier to add new capability or to take advantage of existing functionality by following a well documented API. Fig. 11 shows the new visualization interface where the user is able to customize the window layout to create a multi-plot.

## Figures and Tables

**Table 1:** Experiment planning tools in DAVE

Neutron Cross Section	Table (Fig. 2) Calculator (Sect. 4 A 2)
Self Shielding	Cylindrical/Annular Geometry Slab Geometry
Disk Chopper Spectrometer (DCS)	Experiment Planner (Sect. 4 A 3)
Triple Axis Spectrometer (TAS)	Resolution Calculator Scan Mapper (Sect. 4 A 1)
Hindered Rotor	Diatomic Rigid Rotor Methyl Rotor Coupled Methyl Rotor
General	Number Density Calculator Molecular Weights and Concentrations Neutron Calculator and Units Converter

**Table 2:** Data reduction software modules in DAVE

Facility	Instruments
NIST Center for Neutron Research (NCNR)	Disk Chopper Spectrometer (DCS) Filter Analyzer Neutron Spectrometer (FANS) High Flux Backscattering Spectrometer (HFBS) Neutron Spin Echo Spectrometer (NSE) 4 Triple-Axis Spectrometers (TAS)
Swiss Spallation Neutron Source (SINQ) at the Paul Scherrer Institut (PSI)	FOCUS Time-of-Flight Spectrometer [24] MARS Backscattering Spectrometer [23]
ISIS Pulsed Neutron Source at Rutherford Appleton Laboratory	OSIRIS McStas Simulated Data Reduction [25], [26]
Institut Laue-Langevin (ILL)	Disk Chopper Time-of-Flight Spectrometer (IN5)

## Footnotes

1 Certain commercial equipment, instruments, or materials are identified in this paper to foster understanding. Such identification does not imply recommendation or endorsement by the National Institute of Standards and Technology, nor does it imply that the materials or equipment identified are necessarily the best available for the purpose.
